# Percutaneous cryoablation of chondroblastoma and osteoblastoma in pediatric patients

**DOI:** 10.1186/s13244-021-01036-z

**Published:** 2021-07-27

**Authors:** Elena Serrano, Federico Zarco, Anne E. Gill, C. Matthew Hawkins, Napoleón Macías, Emilio J. Inarejos Clemente, Ferran Torner, Ignasi Barber, Daniel Corominas, Enrique Ladera González, Antonio López-Rueda, Fernando M. Gómez

**Affiliations:** 1 Department of Interventional Radiology, Hospital Clínic Barcelona, Barcelona, Spain; 2 Department of Interventional Radiology, Hospital Sant Joan de Déu, Esplugues de Llobregat, Barcelona, Spain; 3 Department of Interventional Radiology, Children’s Healthcare of Atlanta, Egleston Hospital, Atlanta, Georgia; 4 Department of Radiology, Hospital Sant Joan de Déu, Esplugues de Llobregat, Barcelona, Spain; 5 Pediatric Orthopaedics and Traumatology Department, Hospital Sant Joan de Déu, Esplugues de Llobregat, Barcelona, Spain

**Keywords:** Osteoblastoma, Chondroblastoma, Percutaneous thermal ablation, Radiofrequency ablation, Cryoablation

## Abstract

**Background:**

To review the safety and efficacy of percutaneous cryoablation for the treatment of chondroblastoma and osteoblastoma in the pediatric and adolescent population.

**Materials and methods:**

A retrospective review from 2016 to 2020 was performed to evaluate clinical and imaging response to percutaneous cryoablation in 11 symptomatic patients with diagnosis of chondroblastoma and osteoblastoma treated from two pediatric hospitals with at least 12-month follow-up. Technical success (correct needle placement and potential full coverage of the tumor with the planned ablation zone) and clinical success (relief of the symptoms) were evaluated. The primary objective was to alleviate pain related to the lesion(s). Immediate and late complications were recorded. Patients were followed in clinic and with imaging studies such as MRI or CT for a minimum of 6 months.

**Results:**

A total of 11 patients were included (mean 14 years, age range 9–17; male *n* = 8). Diagnoses were osteoblastoma (*n* = 4) and chondroblastoma (*n* = 7). Locations were proximal humerus (*n* = 1), femur condyle (*n* = 1), and proximal femur (*n* = 1) tibia (*n* = 3), acetabulum (*n* = 3), thoracic vertebra (*n* = 1) and lumbar vertebra (*n* = 1). Cryoablation was technically successful in all patients. Clinical success (cessation of pain) was achieved in all patients. No signs of recurrence were observed on imaging follow-up in any of the patients. One of the patients developed periprocedural right L2–L3 transient radiculopathy as major immediate complication.

**Conclusions:**

Percutaneous image-guided cryoablation can be considered potentially safe and effective treatment for chondroblastoma and osteoblastoma in children and adolescents.

## Keypoints

- Percutaneous cryoablation is a treatment option for bone benign tumors in pediatric population.
- Cryoablation is a safe treatment of chondroblastoma and osteoblastoma in children and adolescents.
- Cryoablation of chondroblastoma and osteoblastoma is effective for pain control in pediatric patients.

## Background

Chondroblastoma and osteoblastoma are rare benign, locally aggressive bone tumors. Despite their low risk of malignant transformation, they can cause local complications such as pain disability and gait disturbances which may significantly impact the patient’s quality of life [1].

Osteoblastoma commonly involves the spine and long bones. The radiographic features of osteoblastoma include an expansile lytic lesion with or without matrix mineralization. Otherwise, most chondroblastomas occur in the long tubular bones and almost invariably involve the epiphysis; they incite inflammatory changes in the surrounding tissues, resulting in pain and decreased range of motion [2].

Surgical resection and curettage are still considered the main treatment for these tumors; however, recurrence rate ranges from 10 to 35% in the case of chondroblastoma and this type of surgical procedure can cause physical and emotional trauma for pediatric patients [3]. Percutaneous ablation represents a safe and effective minimally invasive alternative for treatment of benign musculoskeletal tumors in children [4]. Radiofrequency ablation (RFA) is the most commonly used thermal ablation method and has shown positive and long-standing effects for the local control of osteoblastoma and chondroblastoma [5].

Other thermal percutaneous ablation treatments, such as cryoablation, have been used to treat benign bone tumors [4]. Despite the common use of cryoablation to manage painful bone metastases, its use to treat benign, locally aggressive musculoskeletal lesions remains limited. Compared to cryoablation, RFA is the most commonly used thermal ablation method and has shown positive and long-standing effects for the local control of osteoblastoma and chondroblastoma [5]. However, the ablation zone of RFA can be more difficult to control and there are more permanent complications associated with nerve injury during RFA procedures [6]. Cryoablation has also been demonstrated to be safe and efficacious for the treatment of these types of lesions [7].

The purpose of this retrospective study was to evaluate the safety and efficacy of percutaneous cryoablation minimally invasive treatment option for chondroblastoma and osteoblastoma in children and adolescents.

## Material and methods

### Patients selection

Eleven patients from two academic pediatric centers with chondroblastoma and osteoblastoma were treated with minimally invasive cryoablation procedures between November 2016 and January 2020. The data from the patients’ presentations, procedures and post-procedure courses were retrospectively analyzed. The imaging techniques used for the initial diagnosis were magnetic resonance imaging (MRI) and/or computed tomography (CT). The decision regarding type of treatment was individualized by a multidisciplinary team and discussed with the patient and relatives. Demographic data (patients’ age and gender) and clinical presentation were recorded. Type of tumor, tumor extent (size), location, history of previous treatment/s and number of cryoprobes used for treatment were collected.

### Ethics

The retrospective review of prospectively collected data was approved by the local Clinical Research Ethics Committee (PIC-170-20) and Institutional Review Board.

### Safety and efficacy variables

The complications were recorded and classified as intraoperative (< 24 h), perioperative (< 30 days) and delayed (≥ 30 days) [8]. Complications were also classified according to severity as: 1. mild adverse event (AE): no therapy or non-substantial therapy; 2. moderate AE: moderate escalation of care requiring substantial treatment; 3. severe AE: marked escalation of care, hospital admission or prolongation of existing hospital admission for > 24 h, inpatient transfer from regular floor to intensive care unit; 4. life-threatening or disabling event; 5. patient death [9].

Technical success was defined as the capacity to fully cover the lesion within the ablation zone which was verified on post-procedure imaging and defined as decreased tumor enhancement after contrast administration and/or decrease size of the overall lesion. Primary outcome was clinical success which was defined as resolution of pain, and/or ability to return to daily activities, and/or no need for analgesics after cryoablation.

Radiological response with MRI was achieved if a heterogeneously hyperintense area on T2 was identified in the tumor bed, with no evidence of original tumor. Complete radiological resolution was defined as the entire disappearance of the lesion, without residual alteration of the signal in the bone.

### Technical procedure

**Fig. 1:**
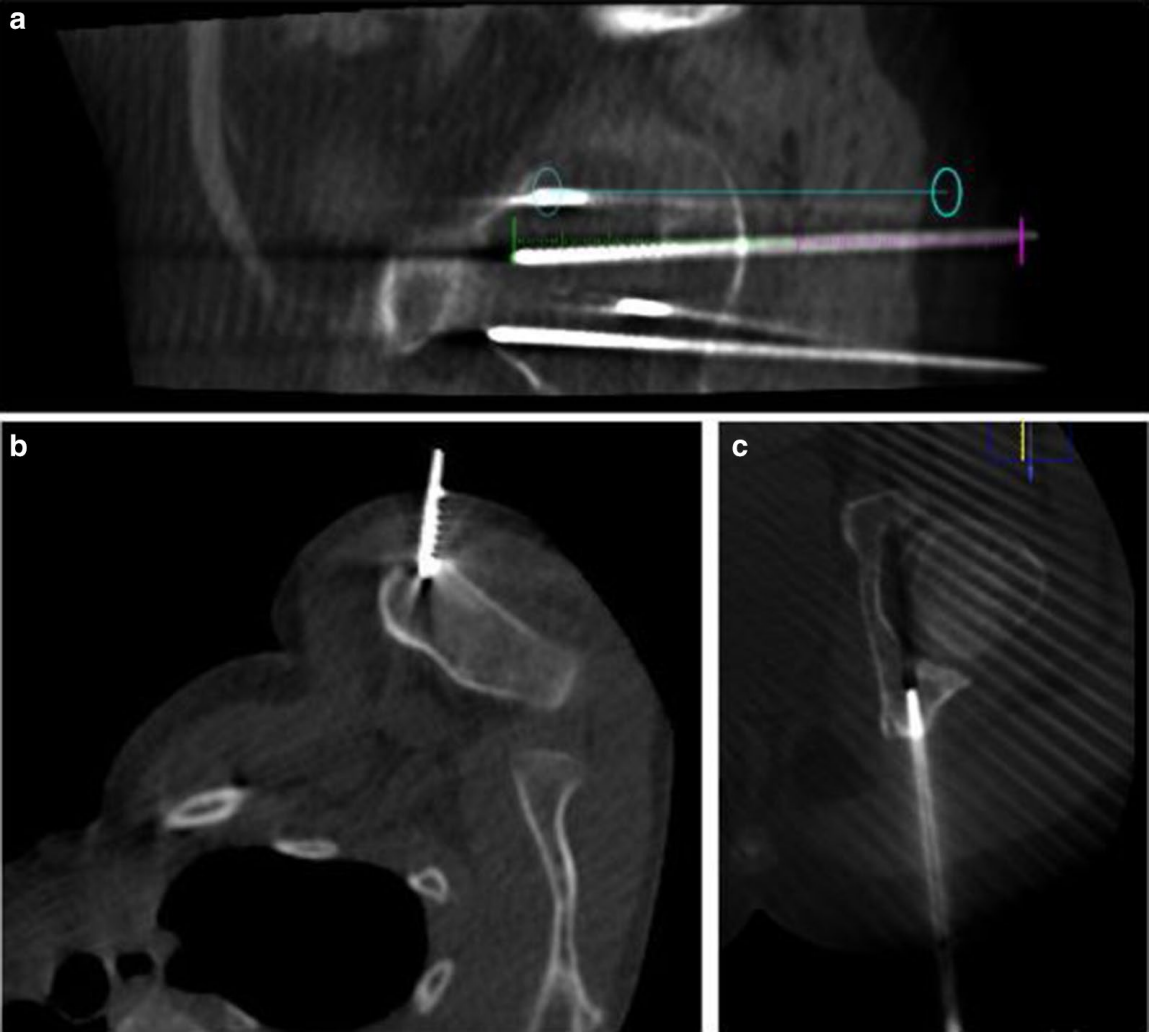
Procedure planification. **a** Live fluoroscopy is projected over a previously acquired three-dimensional data set using cone-beam CT guidance and XperGuide tools. The blue line represents the planned needle course derived by cone-beam CT, which was planned on a separate three-dimensional workstation. **b**, **c** Cryoprobes trajectories placed inside the lesions

Informed consent was obtained from both children and parents before the procedure. All patients underwent general anesthesia. Before each procedure, the percutaneous approach was planned and the best needle trajectory was chosen, avoiding critical structures. Conventional computed tomography or cone beam CT (XperCT/Allura angiography system; Philips, Best, Netherlands and Alphenix angiography system with needle guidance; Canon, Tochigi, Japan) was used for procedural guidance and needle placement during the procedure. Percutaneous cryoablation was performed using Visual-ICECryoablation System (Galil Medical—BTG Arden Hills, Minnesota, USA) equipped with cryoprobes of 15–17 Ga, inserted coaxially through an introducer needle (Powered Bone Access System 10 Gauge, On-control, Arrow or Bone Biopsy System, 14 Gauge, Bonopty, AprioMed). The number and type of cryoprobes were selected based on preoperative imaging with careful attention to the tumor size and morphology. When more than one probe was required, they were placed with a separation of 1.5–2 cm to allow synergistic effect [10] between the probes (Fig. 1).

For the 9 patients treated at one hospital, the cryoablation cycle used included two cycles of freezing (10 min), separated by two thawing phases (10 min, 7 passive and 3 active). In the other center, the cryoablation protocol included two cycles of 10 min freezing separated by two cycles 5 min active thaw.

When needed, insulation techniques were used to prevent damage to adjacent structures such as nerves, skin or bladder. Cross-sectional imaging was performed in the first freezing phase, after 8 min of cooling, to monitor the ablation zone and look for changes in surrounding tissues.

All patients were discharged less than 24 h after the procedure.

### Follow-up protocol

Patients were scheduled for follow-up in the interventional radiology or orthopedic outpatient clinic to assess post-procedural pain and other symptoms 1–6 months after the procedure. Follow-up imaging of the bone tumors was performed within the first 12 months with MRI and/or CT to rule out complications and assess radiological response. MRI was the imaging modality of choice; however, CT was chosen in a patient in whom MRI was contraindicated due to the presence of a cochlear implant.

### Statistical analysis

Descriptive analysis included frequencies and percentages for categorical variables and mean (standard deviation; SD) or median (interquartile range; IQR) for continuous variables. Statistical analyses were performed using the Statistical Package for the Social Sciences software, version 20.0 (SPSS, Chicago, IL, USA).

## Results

A total of 11 sessions of cryoablation were performed in 11 patients (median 14 years, age range 9–17); 8 patients (72%) were male. Four lesions (36%) were osteoblastoma and seven (64%) chondroblastoma. Mean size of the lesions was 20 mm (largest diameter range 8–35 mm). The primary and presenting symptom for all patients was pain. Five patients (45%) were treated for tumor recurrence after initial RFA (*n* = 2) or surgical curettage (*n* = 3). Table 1 summarizes patient demographics, clinical presentation, diagnosis, tumor size, tumor location and any previous treatments. The remainder of patients (*n* = 6) were directly referred to interventional radiology for primary treatment of the benign tumor with cryoablation.

**Table 1:** Patient demographics, clinical presentation, diagnosis, tumor size, tumor location and previous treatments

	Age (years)	Gender	Diagnosis	Location	Max size	Presenting symptom	Previous treatment
#1	14	Male	C	Left acetabulum	8 mm	Pain	None
#2	17	Female	O	Lumbar vertebra	22 mm	Pain	None
#3	12	Male	O	Left acetabulum	15 mm	Pain	RFA
#4	14	Female	C	Left acetabulum	20 mm	Pain	None
#5	13	Male	C	Femur	26 mm	Pain	Curettage
#6	15	Male	C	Femur	35 mm	Pain	None
#7	9	Female	O	Proximal humerus	22 mm	Pain	Curettage
#8	14	Male	O	Thoracic vertebra	20 mm	Pain	RFA
#9	15	Male	C	Tibia	18 mm	Pain	Curettage
#10	14	Male	C	Tibial epiphysis	19 mm	Pain	None
#11	14	Male	C	Tibial epiphysis	16 mm	Pain	None

*C* chondroblastoma, *O* osteoblastoma, *RFA* radiofrequency ablation

**Fig. 2:**
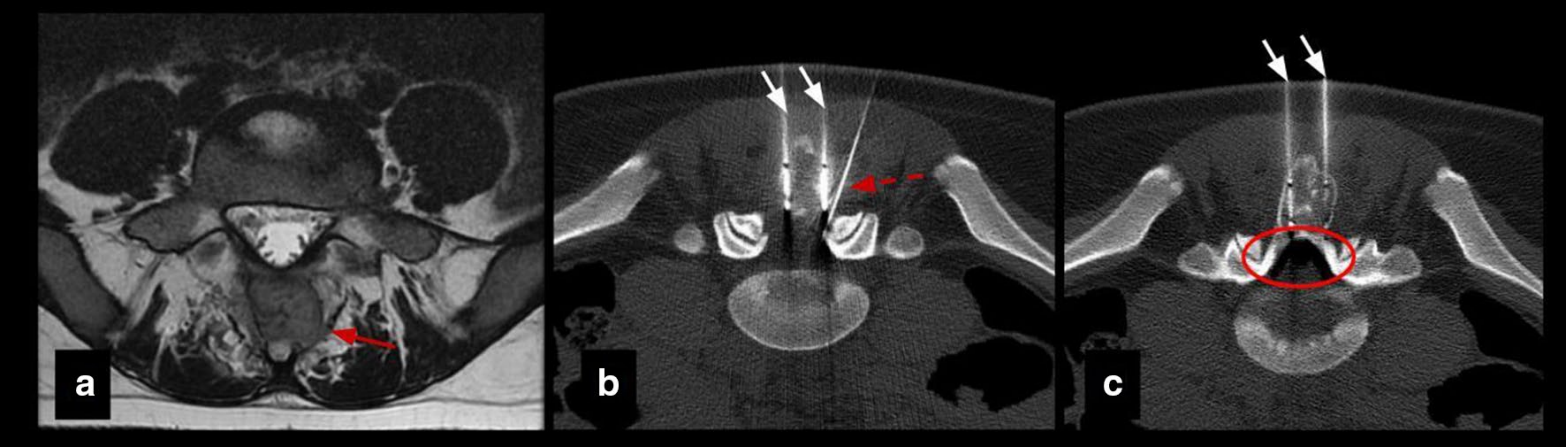
Insulation technique: epidural pneumo-dissection. Osteoblastoma in a 17-y.o patient. **a** Axial T2 MR image shows an expansile lesion in the posterior elements of L5 (red arrow). **b**, **c** Axial CT images show cryoprobes (white arrows) and epidural needle (dashed arrow) for pneumo-dissection system (circle) used to protect L4 and L5 nerve roots

Percutaneous cryoablation was technically successful in all patients (the lesions were able to be accurately targeted and appropriately covered by the ablation zone). Table 2 summarizes procedural and follow-up specifics. Insulation techniques were required in 5 patients (45%). Hydrodissection was carried out in four patients (36%), sterile saline was injected in the joint space in lesions with intra-articular or juxta-articular position to protect the cartilage and in the epidural space to protect spinal cord and nerve roots in vertebral lesions (Fig. 2). Subdermal fluid injection was necessary in one patient (9%) to protect the skin in the treatment of one osteoblastoma of the humerus located superficially.

**Table 2:** Procedural and follow-up specifics

	Number of probes for cryoablation	Insulation technique	Technical success	Immediate complications	Late complications	Time to first outpatient follow-up (weeks)	Pain at first follow-up	Total follow-up (months)	Recurrence	Retreatment
#1	1	None	Yes	None	None	6	No	23	No	None
#2	2	Epidural pneumo dissection	Yes	None	None	5	No	31	No	None
#3	1	Joint hydrodissection	Yes	None	None	1	No	27	No	None
#4	1	Joint hydrodissection	Yes	None	None	8	No	14	No	None
#5	3	Joint hydrodissection	Yes	None	None	2	No	10	No	None
#6	3	None	Yes	None	None	7	No	10	No	None
#7	1	Skin protection	Yes	None	None	3	No	7	No	None
#8	1	Epidural hydrodissection	Yes	Yes	None	4	No, radiculopathy L2–L3	12	No	None
#9	2	None	Yes	None	None	8	No	15	No	None
#10	1	None	Yes	None	6 months bursitis	6	No	10	No	None
#11	1	None	Yes	None	3 months stress fracture	4	No	33	No	None

According to clinical documentation, in the weeks after cryoablation (mean 4.9 weeks; range 1–8 weeks after the cryoablation), there was satisfactory control of pain in all patients. Patients were followed for a median of 17 months (range 12–36 months).

One immediate moderate complication was observed; one of the patients developed periprocedural right L2–L3 transient radiculopathy after the treatment of an osteoblastoma located in the 11th thoracic vertebral body. Two late complications have been documented, one case of tibial stress fracture and one case of bursitis 3 and 6 months after treatment, respectively, although its association with the procedure is not entirely clear. The stress fracture was not at the site of the procedure, and the area of bursitis was adjacent to the access site but not in the ablation zone; thus, these injuries may be related to overuse once the bone tumor pain resolved following cryoablation. One of the patients developed impaired ambulation without pain 6 months after treatment and the MRI showed Osteochondritis dissecans. It has to be highlighted that the patient is a high-performance athlete; therefore, this complication could be due to repetitive trauma during sport [11], so we have not included it as a complication.

There are no imaging signs of recurrence in any of the 11 patients in subsequent imaging tests during the post-procedure follow-up period (median imaging follow-up = 24 months, range 12–36 months).

## Discussion

Previous case reports and case series have been published about cryoablation of osteoblastomas in the adult population. In 2015, percutaneous cryoablation of a large pelvic osteoblastoma recurring after surgery was reported for the first time by Kumasaka et al. [12], with excellent clinical results regarding pain relief and absence of recurrence. Recently, Cazzato et al. [13] reported in 2019 an observational study on ten patients who underwent osteoblastoma cryoablation, some of them on pediatric age (not specified, range 6–54 years), with excellent results and complete pain relief at 1 month in all of the cases.

Poullain et al. [14] have recently reported a case of percutaneous cryoablation of a painful osteoblastoma located in the intertrochanteric region of the proximal femur, with complete resolution of symptoms and disappearance of the edema pattern in MRI follow-up and fat substitution in the peripherical zone of the tumor.

Regarding chondroblastoma cryoablations, Thibaut et al. [15] first reported three adolescent patients with chondroblastoma treated by cryoablation all with excellent clinical results.

**Fig. 3:**
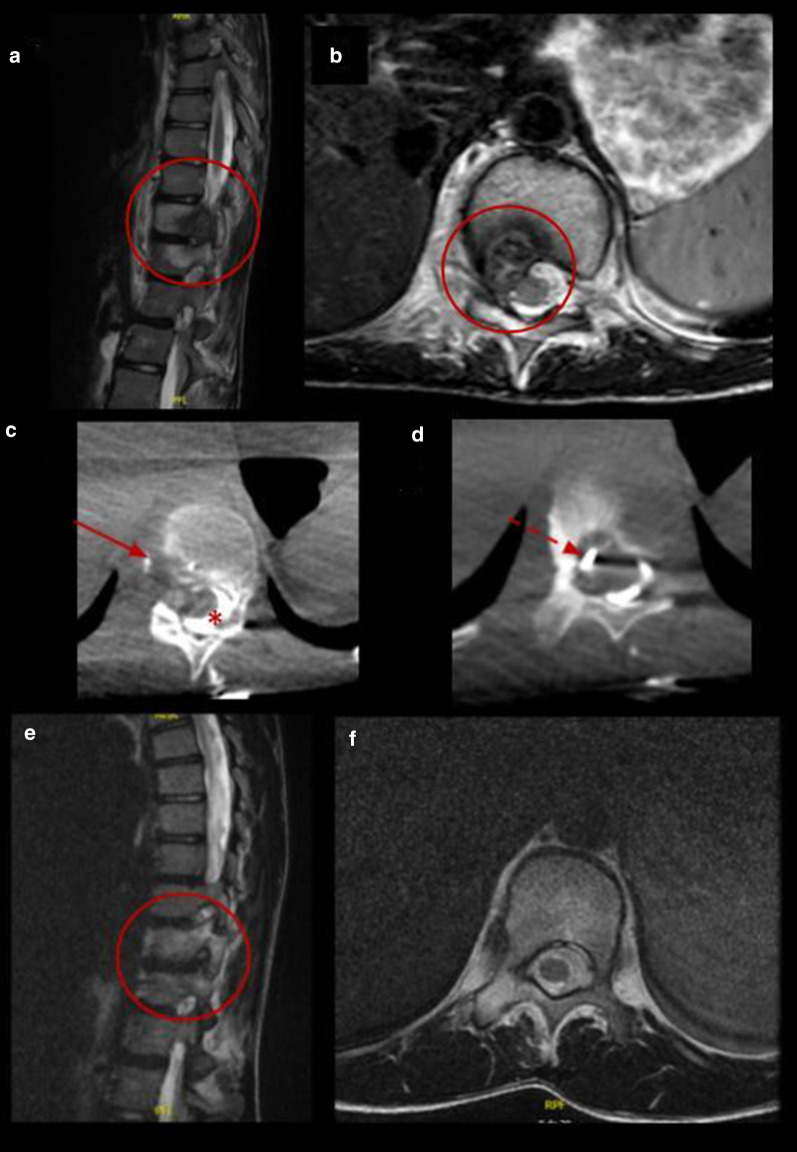
Percutaneous cryoablation of vertebral osteoblastoma in a 14-y.o patient. **a**, **b** Sagittal and axial T2 MR images show a lobulated lesion located in the posterior aspect of the body of the 11th thoracic vertebra (red circle). **c**, **d** Cryoablation of OB (arrows). Cone beam CT axial reconstruction of the cryoprobe and epidural hydrodissection (asterisk). **e**, **f** Follow-up MRI 12 months after procedure with complete resolution of the lesion (red circle)

**Fig. 4:**
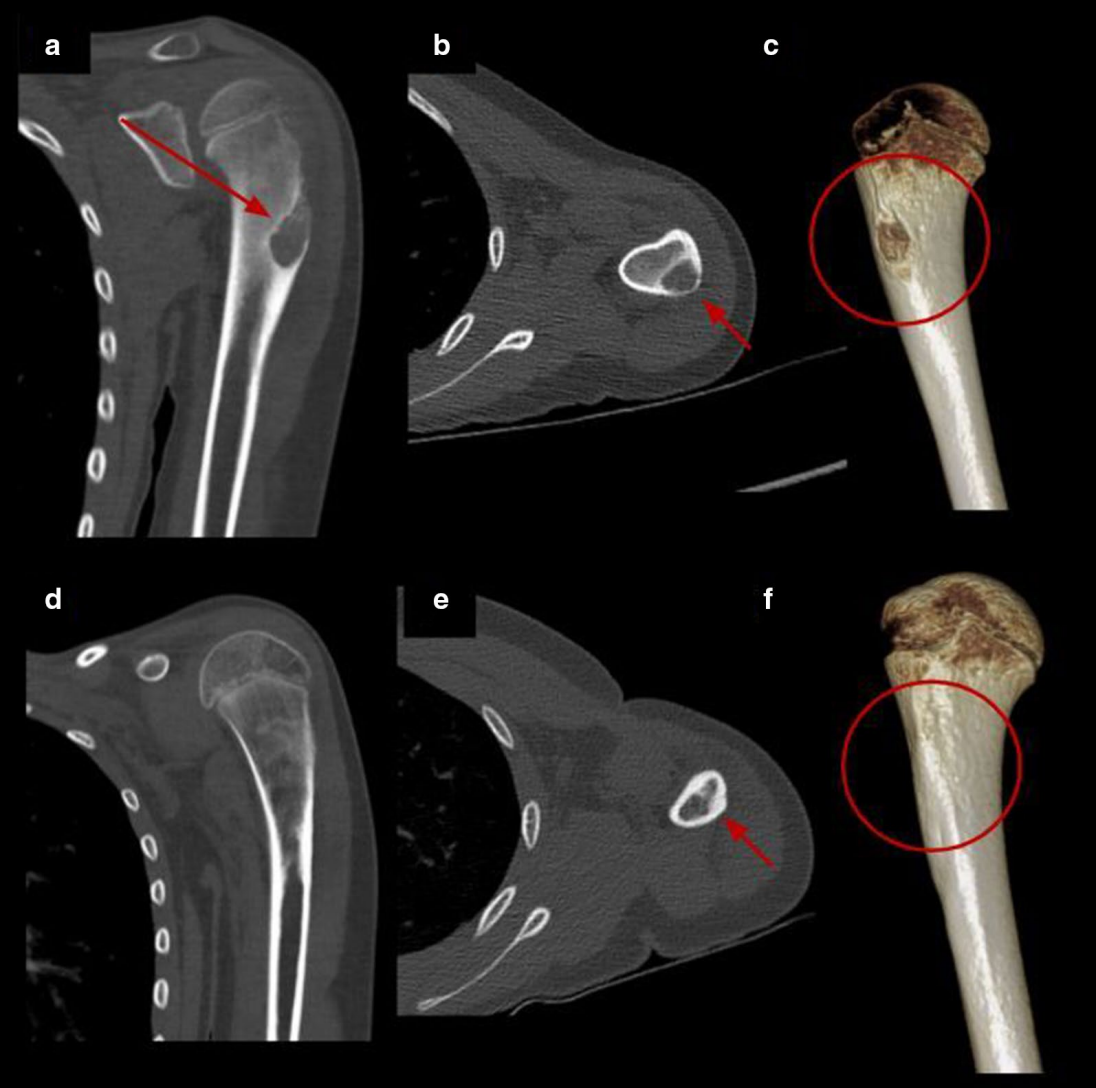
Percutaneous cryoablation of humeral osteoblastoma in a 9-y.o patient. **a–c** Coronal, axial CT images and volume rendering reconstruction of the left humerus show a lytic lesion consistent with osteoblastoma (red arrow and red circle). **d–f** Control CT 7 months after treatment with complete resolution of the lesion (red arrow and red circle)

**Fig. 5:**
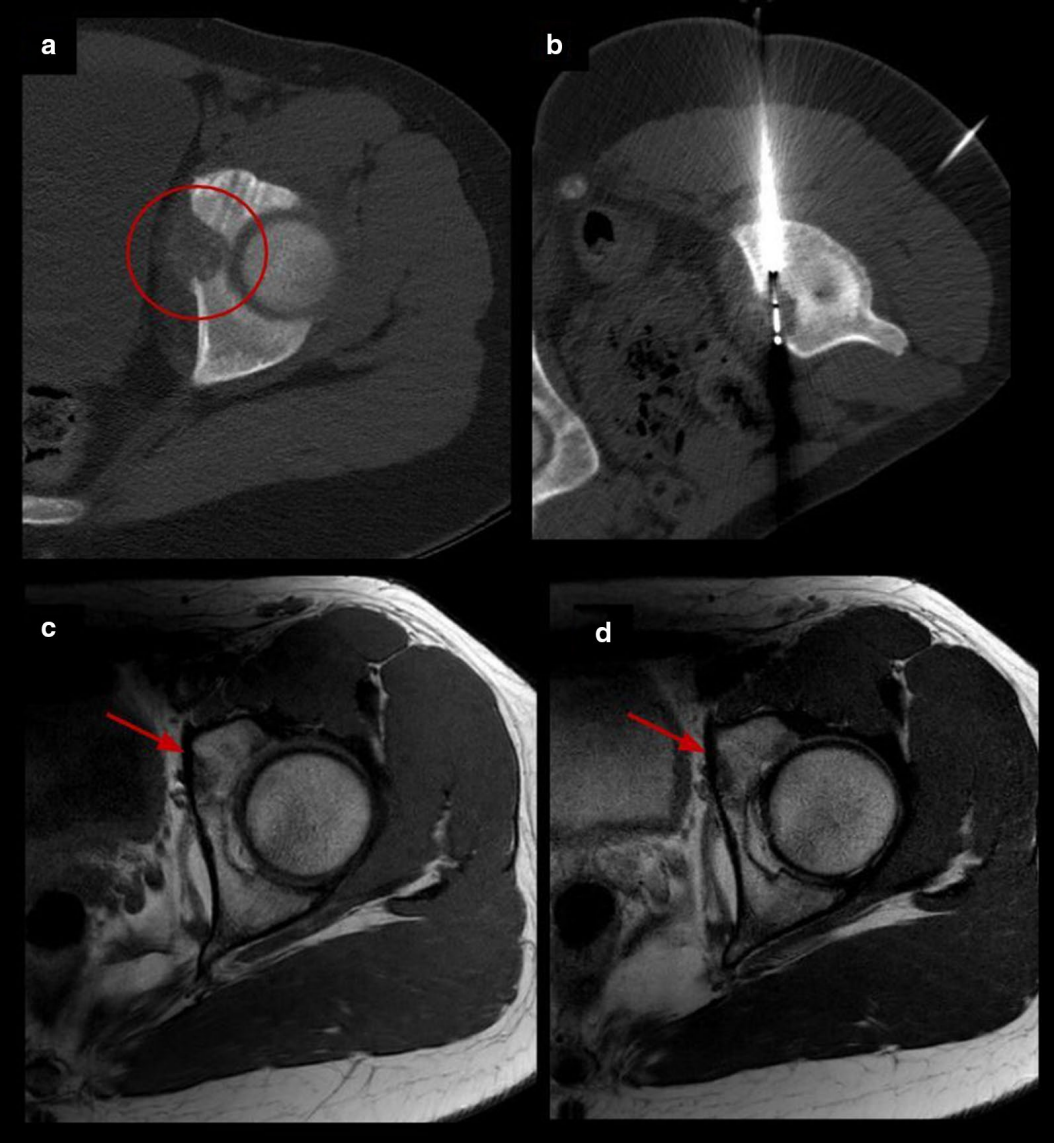
Acetabulum osteoblastoma cryoablation. **a** Axial CT images demonstrating an expansive osteolytic tumor with partial matrix mineralization in the left acetabulum corresponding to osteoblastoma (circle). **b** Axial CT exhibiting one cryoprobe placed within the nidus. **c, d** Follow-up MRI images 15 months after treatment with complete resolution of the lesion (arrows)

**Fig. 6:**
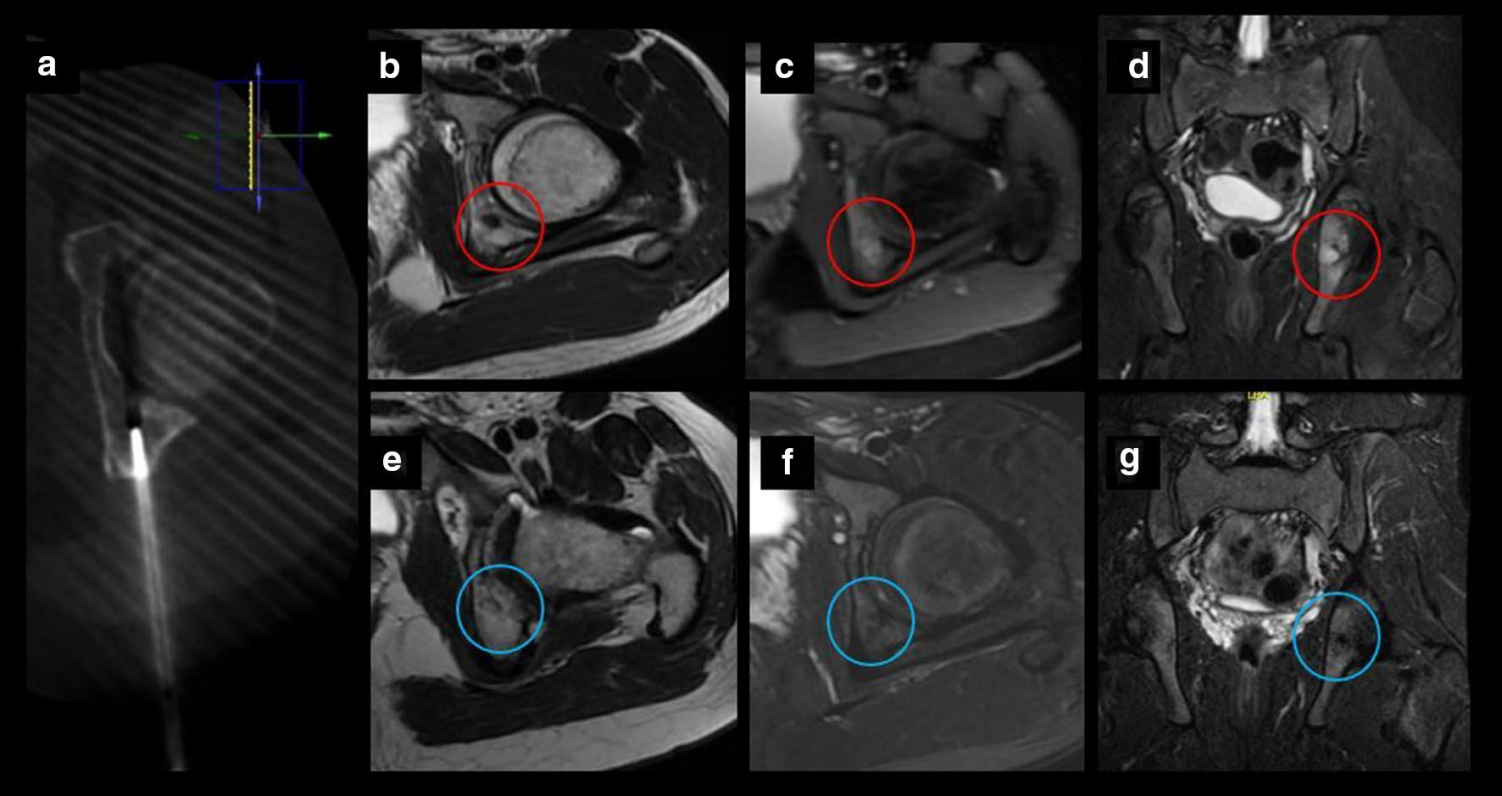
Chondroblastoma in a 14-y.o patient. **a** Axial CT exhibiting one cryoprobe placed within the lesion. **b–d** MRI images demonstrating a small tumor in the left posterior acetabulum corresponding to chondroblastoma (red circle). **e–g** Follow-up MRI images 20 months after treatment with no evidence of recurrence (blue circle)

Our retrospective study shows a case series of eleven pediatric and adolescent patients with chondroblastoma or osteoblastoma in which image-guided percutaneous cryoablation achieved complete pain control. Six patients were treated with cryoablation only as the primary and only intervention to treat their pain, while a minority of patients five patients were referred for cryoablation following incomplete response of the lesions after RFA or surgical curettage. No signs or symptoms of recurrence were detected in clinical and imaging follow-up. Furthermore, complete radiological tumor resolution was observed in osteoblastoma cases with complete disappearance of the tumor and no residual bony changes within the first 2 years after treatment (Figs. 3, 4, 5 and 6).

The medical literature cites an overall complication rate of 9.1% associated with cryoablation of bone lesions and a major complication rate of 2.5%. Secondary fracture is the most common major complication of bone tumor cryoablation, occurring in approximately 1% of patients [16]. Some of the immediate complications of cryoablation are related to thermal damage to nearby structures. Different techniques to protect the critical structures at risk of being injured have been described in the medical literature; furthermore, the insulating techniques improve the safety of the procedure for complex lesions in challenging locations [17]. In this series, air and hydrodissection were used in 5 patients to protect adjacent cartilage from the ablation zone used to treat intra- or juxta-articular lesions. In this series, one immediate moderate complication (according to the Society of Interventional Radiology (SIR) [9] has been observed: periprocedural, right L2–L3 transient radiculopathy after the treatment of an osteoblastoma located in the body of the 11th thoracic vertebra. Despite the injection of diluted contrast in the epidural space and the neurophysiological and thermal control during the procedure (that showed no alterations), the patient experienced pain and difficulty lifting the right foot after the intervention without sensory deficit. Due to restrictions on in person clinic visits secondary to SARS CoV2 pandemic, telephone consultation 4 months after treatment was made. During the telemedicine appointment, complete resolution of symptoms was confirmed. Neurological consultation 12 months after the intervention confirmed the resolution of radiculopathy symptoms with normal physical examination, normal sensation and motor control, as well as complete resolution of the lesion on the follow-up thoracic spine MRI.

The two delayed moderate complications included a stress fracture and a bursitis. The stress fracture happened 4 weeks after the procedure. This complication was clinically manifested as reappearance of pain, which was resolved once the fracture was treated. The bursitis of the knee manifested 6 months after treatment near the access site (different location than presenting symptoms of the chondroblastoma). MRI showed stable post-ablation changes with inflammation in the anteromedial knee. As the patient still complained, a biopsy of the lesion was made to confirm that the pain was not associated with the recurrence of the bony lesion. So, as neither of the complications were located in the ablation area, the authors speculate the bursitis and stress fracture could be secondary injuries related to overuse after pain had improved following cryoablation.

No signs of bony tumor recurrence on follow-up imaging studies were detected. Cazzato et al. [13] recently reported 10 cases of osteoblastoma treated with percutaneous cryoablation; findings of treatment success on follow-up imaging included variable degrees of bone remineralization noted on successive CT studies and no contrast enhancement of the osteoblastoma on the MRI. Complete resolution of the osteoblastoma on follow-up imaging, without residual or fibrotic changes, described in this retrospective study, is a very promising finding but has not been previously described. This phenomenon will need further evaluation in prospective studies with larger cohorts. Long-term radiological follow-up is necessary to discover if chondroblastoma will show similar imaging results as osteoblastoma after cryoablation.

In accordance with previous reports, this study supports the advantages of cryoablation over other techniques. First, cryoablation could be monitored during the procedure with intermittent non-contrast CT, ultrasonography or MRI, since it is possible to visualize the ice ball corresponding to the future zone of necrosis. This allows for better visualization of ablation effects on adjacent soft tissues [18]. Moreover, it is possible to use multiple cryoprobes simultaneously, which allows for larger ablation zones and synergistic effects of the ice ball to improve coverage of the tumor. Cryoablation, in contrast to RFA, is useful in sclerotic injuries [19]. The anesthetic effect of ice makes it a well-tolerated technique. Finally, compared to surgery, cryoablation can be performed as an outpatient, limits scarring, decreases time of rehabilitation and may reduce morbidity.

Some limitations of our analysis should be considered: first, its retrospective nature and the relatively small sample size. The absence of long-term follow-up could have offered additional information. The lack of a quantitative measurement scale (as visual analogue scale) is another limitation that would have helped to document the severity of the pain before and its objective evolution after the treatment. One more limitation is related to the lack of standardization of image follow-up after the procedure, being necessary the development of a protocol in order to know the prevalence of complete resolution after cryoablation and the evolution of the inflammatory changes after the treatment.

## Conclusions

Percutaneous cryoablation appears to be a safe and clinically efficacious therapeutic option in the treatment of chondroblastoma and osteoblastoma in children and adolescents.

## Data Availability

The datasets used and analyzed during the current study are available from the corresponding author upon reasonable request.

## Abbreviations

AE: Adverse event
CT: Computed tomography
MRI: Magnetic resonance imaging
RFA: Radiofrequency ablation
